# An unusual case of seronegative, 16S PCR positive *Brucella* infection

**DOI:** 10.1099/jmmcr.0.005050

**Published:** 2016-10-27

**Authors:** Binutha Bharathan, Lucy Backhouse, David Rawat, Sandhia Naik, Michael Millar

**Affiliations:** ^1^​ Department of Infection, Bart’s Health, 80 Newark Street, Whitechapel, London, E1 2ES, UK; ^2^​ Department of Paediatrics, Bart’s Health, Royal London Hospital, Whitechapel Road, London, E1 1BB, UK

**Keywords:** *Brucella*, splenic abscesses, pleural effusion, 16S PCR, brucella serology

## Abstract

**Introduction:**

*Brucella* is a zoonotic infection commonly diagnosed by isolation of the organism from blood culture or positive serological testing. It is an uncommon cause of a pyrexia of unknown origin in the United Kingdom.

**Case presentation:**

We describe the case of a 14-year-old girl with no history of travel who presented with pyrexia, weight loss, arthralgia, multiple splenic abscesses and a subsequent pleural effusion, the latter of which isolated a *Brucella* species on 16S rRNA PCR. The patient responded well to initiation of treatment for brucellosis and on repeat imaging, after 3 months, the splenic abscesses had resolved.

**Conclusion:**

This unique case demonstrates uncommon complications of brucellosis and the challenges of diagnosing the organism, the latter of which can be alleviated by the utilization of molecularbased technologies. This patient had a negative serology result for brucellosis, which highlights the need to interpret serology results with caution in non-endemic regions for brucellosis.

## Introduction

Brucellosis is a zoonotic infection characteristically presenting with an undulating fever. Thirty per cent of infections can result in localized infection (Aygen *et al.*, 2002; Hasanjani Roushan *et al.*, 2004) of which splenic abscesses represent a small minority of that. In the paediatric group, there are only two documented cases (Vallego *et al.*, 1996; Parande *et al.*, 2010).

We describe a rare case of splenic and pulmonary brucellosis in a patient with no recent travel. This interesting case demonstrates the complex nature of diagnosing brucellosis and the benefits of molecular testing in a disease normally exclusively diagnosed by serology and blood culture.

## Case report

A 14-year-old British born girl presented with a 1 month history of fever, weight loss, lethargy, arthralgia and diarrhoea. She had been investigated a month previously for an iron deficiency anaemia with no cause found. Travel history consisted of a trip to Israel 2 years ago.

Upon examination, she was pyrexial (38.5 °C) and tachycardic with a soft systolic murmur. There was a palpable splenic tip below the left costal margin.

## Investigations

**Fig. 1.**
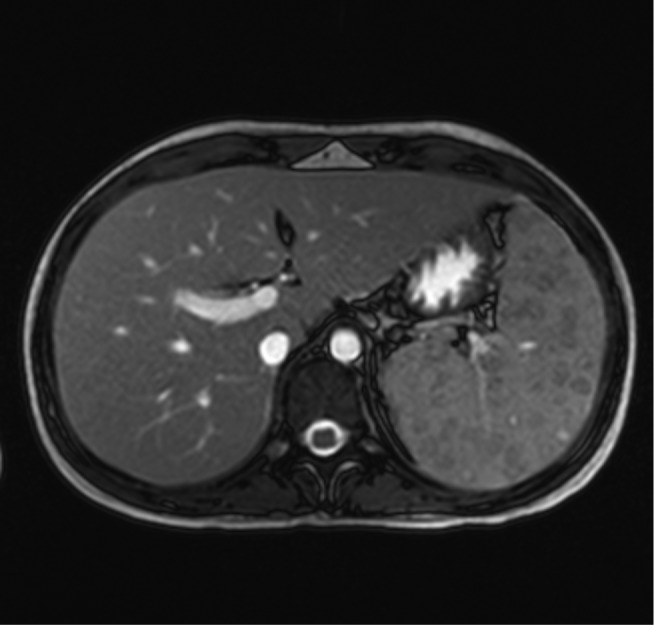
MRI of the abdomen demonstrating multiple hypogenic splenic abscesses.

Bloods demonstrated haemoglobin of 6.1 g dl^−1^, mean cell volume of 72 fl, erythrocyte sedimentation rate of 73 mm h^−1^ and C-reactive protein of 149 mg L^−1^. All other blood parameters were normal. A presumptive diagnosis of inflammatory bowel disease was made. Upper and lower gastro-intestinal endoscopies were unremarkable whereas a magnetic resonance imaging (MRI) of the abdomen showed splenomegaly of 14.8 cm with multiple small, hypogenic splenic abscesses (Fig. 1).

A total of four sets of blood cultures taken over a period of 5 days were negative. The initial antibiotic regimen of ciprofloxacin 500 mg bd was changed to ceftriaxone 2 g od and flucloxacillin 1 g qds on day 7 in order to broaden cover for a potential salmonella or staphylococcal infection.

## Diagnosis

One month into admission, the patient remained pyrexial despite antimicrobial therapy. A transthoracic echocardiogram was negative and a repeat ultrasound of her spleen showed no change in the size of the abscesses, resulting in consideration of a splenec tomy. The patient subsequently developed a left-sided pleural effusion. Pleural aspirate was negative on culture and for acid-fast bacilli but was positive for a *Brucella* species following 16S PCR. Of note, serology was negative using the Brucellacapt immunocapture assay.

## Treatment

Treatment consisted of a 12-week course of rifampicin and doxycycline in conjunction with the addition of gentamicin for the first 14 days.

## Outcome and follow-up

The patient had a good clinical response with complete regression of her splenic abscesses on follow-up ultrasound imaging. In the absence of any recent foreign travel, the case was notified to Public Health of England as a probable UK acquisition.

## Discussion

Brucellosis is caused by small, non-motile Gram-negative coccobacilli. The four species of *Brucella* that are pathogenic to humans are *Brucella melitensis, Brucella abortus*, *Brucella suis* and *Brucella canis* (Franco *et al.*, 2007; Mansur *et al.*, 2008). Infection can be acquired from inhalation of aerosolized bacteria or the consumption of products such as unpasteurized milk and cheese or meats from infected animals (Aygen *et al.*, 2002; Franco *et al.*, 2007). Brucellosis is endemic to areas including the Mediterranean Basin and Middle East. In the UK, an average of 10 cases are reported each year, nearly all of which are acquired from abroad (Health Protection Agency, October 2013).

This case demonstrates the complexity of obtaining a diagnosis of brucellosis whereupon multiple sets of blood cultures are culture negative. This may reflect prior antimicrobial treatment with ceftriaxone and ciprofloxacin, both of which have activity against *Brucella* (Falagas & Bliziotis, 2006; Baykam *et al.*, 2004). In addition, the detection of *Brucella* from blood cultures has previously been demonstrated to have low sensitivity in focal and chronic infections (Azira, 1996).

In the presence of this low yield from blood cultures, serology has become the mainstay of diagnosis with the standard tube agglutination (STA) test being used worldwide as the gold standard for acute brucellosis. A titre value of 1 : 80 in non-endemic regions and 1 : 320 in endemic regions is regarded as a positive threshold for the diagnosis of brucellosis (Christopher *et al.*, 2010). A fourfold increase in titre from the acute to convalescent sample is considered diagnostic. In regions of high seropositivity, isolated low-level STA titres can be difficult to interpret. The ELISA test measuring IgA, IgM and IgG has demonstrated higher sensitivity in the detection of very acute and chronic brucellosis than the STA, although some studies suggest that the overall specificity is lower (Mantur *et al.*, 2010). The Brucellacapt, an immunocapture agglutination test, has the advantage over the aforementioned tests of detecting both non-agglutinating and agglutinating antibodies. It has been shown in studies to detect higher titre values in acute and evolving infections than the STA or Coombs test (Orduña *et al.*, 2000), possibly due to its ability to detect IgM, IgG and IgA and blocking antibodies. The Brucellacapt has shown good correlation with the Coombs test with superior sensitivity to the STA (Casao *et al.*, 2004). The Brucellacapt and the Coombs test are deemed superior serology tests for prolonged active infection and relapsing disease (Bosilkovski *et al.*, 2010). 

In the UK, the Brucellacapt test has superseded the STA for diagnosing *Brucella* due to its ability to detect both acute and chronic infections (Baykam *et al.*, 2004). However, the variable sensitivity of serology tests, especially in non-endemic settings (Fadeel *et al.*, 2011), highlights the need for caution in advocating the widespread use of serology tests alone in diagnosing focal brucellosis.

In the present case, *Brucella* was identified from a pleural fluid sample using broad-spectrum 16S rRNA gene PCR. The benefit of 16S PCR for the identification of bacterial pathogens from pleural fluid when compared to culture alone has been previously reported (Saglani *et al.*, 2005). It has also been proposed that PCR assays are more sensitive at detecting relapses in brucellosis when compared to serological testing (Mitka *et al.*, 2007). At the present time, 16S rRNA PCR has the ability to accurately identify to a genus level but, as in our case, has difficulty speciating *Brucella*. Nevertheless, the current case demonstrates the advantages of utilizing 16S PCR methodology in situations of diagnostic uncertainty and, to the author’s knowledge, represents the first isolation of *Brucella *from a pleural fluid sample.

Splenic abscesses in the context of brucellosis are rare with an incidence reported to be approximately 2–3 % and predominantly associated with chronic hepatosplenic brucellosis (Ariza *et al.*, 2001). Medical treatment alone can be successful as in the aforementioned case but surgical intervention is often required (Solera *et al.*, 1997). Respiratory involvement in brucellosis is also a rare occurrence as demonstrated by Pourbagher *et al*. (2006) who reported the presence of pleural effusions in 2.8 % of patients. Consequently, this case demonstrates the presence of two rare complications arising from brucellosis in a single patient.

Treatment of brucellosis historically consists of dual therapy with rifampicin and doxycycline being advocated by the World Health Organisation for the treatment of uncomplicated cases. A systematic review demonstrated an aminoglycoside for 2 weeks with rifampicin and doxycycline for at least 12 weeks to be superior to standard dual therapy (Skalsky *et al.*, 2008).

The difficulty in diagnosing brucellosis has been consistently reported. This present case adds to this literature by demonstrating the importance of employing molecular techniques in the investigation of unusual pathogens such as *Brucella* from sterile samples. It is the subsequent opinion of the authors that molecular testing for the detection of acute infection and relapses with brucellosis should be encouraged.
